# Quantitative Proteomics Reveals Substrate-Specific Metabolic Adaptations for *n*-Alkane and Branched Alkane Degradation in *Dietzia* sp. CN-3

**DOI:** 10.3390/microorganisms14092100

**Published:** 2026-09-19

**Authors:** Weiwei Chen, Xin Zhang, Nian Liu, Jun Min, Shiwei Cheng

**Affiliations:** 1 School of Life Sciences, Ludong University, Yantai 264025, China; wwchen@ldu.edu.cn (W.C.); 15192081793@163.com (X.Z.); lnian0426@163.com (N.L.); 2 Yantai Institute of Coastal Zone Research, Chinese Academy of Sciences, Yantai 264003, China; jmin@yic.ac.cn

**Keywords:** *n*-alkane, pristane, *Dietzia*, quantitative proteomics, terminal oxidation, subterminal oxidation

## Abstract

Although hydrocarbon-degrading bacteria play a critical role in petroleum bioremediation, the substrate-specific metabolic pathways and adaptive mechanisms that underlie their competitive dominance remain poorly understood. *Dietzia* sp. CN-3, a salt-tolerant bacterium capable of utilizing both linear and branched alkanes, is an ideal model for investigating these regulatory networks. Here, we performed data-independent acquisition (DIA)-based quantitative proteomics to compare the proteomic landscapes of strain CN-3 grown on *n*-hexadecane (C_16_), pristane, and glucose. During growth on C_16_, strain CN-3 expressed a proposed terminal and subterminal oxidation pathway converting *n*-alkanes to acyl-CoA derivatives, involving AlkB and CYP153 hydroxylases, alcohol/aldehyde dehydrogenases, Baeyer–Villiger monooxygenases, and esterases. In contrast, pristane induced an alternative terminal oxidation pathway along with a substantially expanded repertoire of fatty acid β-oxidation enzymes to overcome steric hindrance. Functional heterologous expression of *alkB* in *Pseudomonas fluorescens* KOB2Δ1 restored growth on C_12_–C_16_ *n*-alkanes and enhanced the growth of the pCom8-alkB recombinant on C_28_ (μ_max_ = 0.065 d^−1^, OD_max_ = 0.333), confirming its role in medium- to long-chain alkane utilization. Our findings provide a systematic proteomic framework for understanding alkane oxidation in *Dietzia*, and offer mechanistic insights into the metabolic strategies that potentially drive strain CN-3’s adaptability in hydrocarbon-contaminated environments.

## 1. Introduction

Petroleum hydrocarbons remain one of the most pervasive environmental contaminants worldwide, with alkanes constituting the predominant fraction (20–50%) of crude oil and its refined products [1,2]. The extensive exploitation, transportation, and accidental release of petroleum have led to severe soil and marine pollution, posing persistent threats to ecosystem integrity and human health [3,4]. Of the various physical and chemical remediation strategies currently employed (e.g., incineration, thermal desorption, chemical oxidation, surfactant-based washing), microbial degradation is widely recognized as the most economically viable and environmentally friendly approach for mitigating hydrocarbon contamination [2,4,5]. This biological process is driven by diverse hydrocarbon-degrading microorganisms that employ specialized enzyme systems to oxidize and assimilate alkanes. Consequently, elucidating the molecular mechanisms underlying microbial alkane metabolism is of both fundamental scientific interest and practical biotechnological significance.

Alkanes are structurally categorized into three major classes: linear (*n*-alkanes), branched (iso-alkanes), and cyclic alkanes (cycloalkanes). Over the past decades, *n*-alkanes have been relatively well characterized with respect to strain isolation, degradation capabilities, functional genes, and enzymatic mechanisms involved in their catabolism [4,6]. Branched-chain alkanes, such as pristane (2,6,10,14-tetramethylpentadecane) and phytane, which are isoprenoid hydrocarbons widely distributed in the biosphere, represent a substantially more recalcitrant class of hydrocarbons [2,7,8]. Their methyl branches impose steric hindrance that obstructs conventional enzymatic attack, often requiring auxiliary enzyme systems. To date, only a limited number of bacterial strains have been reported to degrade pristane, including *Alcanivorax* spp. [9], *Rhodococcus* spp. [10], and *Dietzia* spp. [8], yet the enzymatic machinery and regulatory pathways involved remain largely unidentified.

Among the limited bacterial genera capable of degrading both *n*-alkanes and branched alkanes, the genus *Dietzia* has attracted increasing attention due to its remarkable catabolic versatility and exceptional environmental adaptability. Members of this genus are frequently isolated from hydrocarbon-contaminated environments, including oil fields, deep-sea sediments, petroleum refinery effluents, and even extreme habitats [11,12]. The substrate range of *Dietzia* is remarkably broad, spanning C_6_–C_30_ *n*-alkanes in strain E1 [13], C_6_–C_40_ *n*-alkanes, aromatics, and crude oil in strain DQ12-45-1b [14], and C_12_–C_36_ *n*-alkanes, aromatics, crude oil, and branched alkanes (pristane and phytane) in strain CN-3 [8]. Furthermore, *Dietzia* acts as a key partner in petroleum-degrading consortia, cooperating with other degraders (e.g., *Acinetobacter*) and non-degraders such as *Pseudomonas* to form syntrophic networks that enhance bioremediation [12,15,16]. This unique catabolic versatility renders *Dietzia* an ideal model for investigating the mechanistic distinctions between *n*-alkane and branched alkane degradation.

Aerobic alkane degradation, which employs O_2_ as the terminal electron acceptor and reactant for substrate activation, represents the primary rapid response to oil spills in surface environments. This process begins with alkane transport across the cell membrane, followed by bio-oxidation—terminal or subterminal hydroxylation, dehydrogenation, and finally fatty acid β-oxidation—catalyzed by enzymes such as hydroxylases, dehydrogenases, esterases, and hydratases [17,18]. For *n*-alkanes, two major degradation pathways have been established: terminal and subterminal oxidation. The terminal oxidation pathway, which proceeds via successive oxidation of the terminal methyl group to yield a corresponding fatty acid, has been extensively characterized at enzymatic and genetic levels [19,20]. However, the subterminal oxidation pathway, which hydroxylates an internal carbon and subsequently cleaves the product to an alcohol and a fatty acid via ketone and ester intermediates, has been characterized in certain organisms such as *Alcanivorax* and *Acinetobacter* [9,10,21], but remains largely unexplored in *Dietzia*. Compared with *n*-alkanes, the aerobic oxidation of branched alkanes such as pristane and phytane remains much less understood. Although a terminal oxidation pathway has been proposed, the specific hydroxylases and downstream enzymes involved in the catabolism of these compounds have not yet been systematically identified.

The initial hydroxylation step, which determines the substrate specificity and overall degradation efficiency, is catalyzed by distinct classes of alkane monooxygenases. For short- and middle-chain *n*-alkanes, several terminal hydroxylases have been well documented, including the integral-membrane alkane monooxygenase AlkB, the AlkM homolog, and the cytochrome P450 family [22,23,24]. Specifically, AlkB is induced by C_12_–C_16_ *n*-alkanes in *Alcanivorax hongdengensis* strain A-11-3 [22], while pristane and phytane induce AlkB2 and P450a in *Alcanivorax borkumensis* SK2 [9,23]. In *Dietzia* sp. DQ12-45-1b, CYP153 acts on C_8_–C_10_ *n*-alkanes, whereas AlkW targets C_12_–C_32_ alkanes [24]. To date, only two long-chain alkane hydroxylases have been characterized: LadA, which oxidizes C_15_–C_36_ *n*-alkanes in *Geobacillus thermodenitrificans* NG80-2 [25], and AlmA, identified in *Acinetobacter* spp. and *Alcanivorax* spp., which targets >C_32_ alkanes [18,26].

Proteomic profiling directly captures the functional state of cellular metabolism and provides complementary insights to genomics and transcriptomics for understanding physiological responses of alkane-degrading bacteria [9,27,28]. The first proteomic investigation of alkane metabolism was conducted on *A. borkumensis* SK2, a strain equipped with both AlkB and cytochrome P450 alkane hydroxylase systems [27]. In *Pseudomonas aeruginosa* SJTD-1, which harbors both AlkB1, AlkB2 and P450 alkane hydroxylase systems, the *alkB2* gene was found to be preferentially activated over its P450 counterpart [28]. In *Dietzia*, however, the activity of alkane hydroxylase genes (*alkB* and *P450*) has been assessed primarily through RT-qPCR or transcriptomic analyses, rather than at the proteomic level [17,24,29]. To address this gap, we performed a data-independent acquisition (DIA)-based quantitative proteomics analysis of *Dietzia* sp. CN-3 grown on *n*-hexadecane (C_16_) and pristane, respectively, aiming to systematically compare the substrate-specific proteomic responses to linear and branched alkanes. This study is designed to identify differentially expressed proteins and related metabolic pathways, thereby providing a protein-level foundation for understanding the metabolic versatility of this environmentally important hydrocarbon-degrading bacterium.

## 2. Materials and Methods

### 2.1. Bacterial Strains and Growth Conditions

The bacterial strains and plasmids used in this work are listed in Table 1. Strain CN-3, a salt-tolerant bacterium previously isolated from petroleum-contaminated sediment in Bohai Bay, China [8], was able to grow on C_12_–C_36_ *n*-alkanes, branched alkanes (pristane and phytane) and crude oil as the sole carbon sources. The major pollutants at the isolation site include total petroleum hydrocarbons, *n*-alkanes, polycyclic aromatic hydrocarbons, and heavy metals [16]. *Pseudomonas fluorescens* KOB2Δ1, an *alkB* knockout mutant of strain CHA0 that is defective in C_10_–C_16_ *n*-alkane utilization, is widely employed as a heterologous expression host for evaluating alkane hydroxylase activity [30,31]. *D.* sp. CN-3, as well as *P. fluorescens* KOB2Δ1 and its recombinant transformants, were cultivated at 30 °C in LB broth or mineral salts medium (MSM) containing 0.1% (*v*/*v*) *n*-alkanes as the sole carbon sources. All liquid cultures were incubated aerobically with shaking at 180 rpm in a ZQPL-200 shaker (Tianjin Labotery Instrument Equipment Co., Ltd., Tianjin, China). DNA transformation and plasmid propagation were performed using *Escherichia coli* DH5α grown in LB medium at 37 °C with shaking at 180 rpm. For maintenance of recombinant plasmids, gentamicin was added to the culture media at final concentrations of 100 µg mL^−1^ for *P. fluorescens* KOB2Δ1 transformants and 10 µg mL^−1^ for *E. coli* DH5α.

### 2.2. Bacteria Cultivation and Protein Preparation

Cells of CN-3 were harvested at the mid-exponential phase from cultures grown with C_16_, glucose, or pristane as the sole carbon source, with incubation periods of 1, 2, and 14 d, respectively. These incubation periods were determined based on growth curves established in our previous study [29] by monitoring OD_600_ over time, ensuring comparable physiological states across all conditions. In addition, the degradation rates of C_16_ and pristane by CN-3 were obtained from our previous study [29], in which the GC-MS method is described in detail. Four independent biological replicates were included for each condition. Bacterial cells were pelleted via centrifugation at 4 °C and 8000 rpm for 10 min, immediately snap-frozen in liquid nitrogen for 20 min, and then sent to Shanghai Majorbio Bio-pharm Technology Co., Ltd. (Shanghai, China) for further analyses. A schematic overview of the experimental workflow is provided in Supplementary Figure S1.

Cellular pellets were resuspended in a lysis buffer composed of 8 M urea and 1% SDS, supplemented with a protease inhibitor cocktail to prevent proteolytic degradation. The suspensions were pre-cooled in liquid nitrogen, disrupted using a Wonbio-96D cryogenic grinder (Shanghai Wonbio Biotechnology Co., Ltd., Shanghai, China) through three grinding cycles (each lasting 180 s), and then further sonicated using a SCIENTZ08-IIIC non-contact ultrasonic cell disruptor (Ningbo Scientz Biotechnology Co., Ltd., Ningbo, China) for 30 min. Insoluble debris was removed by centrifugation at 16,000× *g* and 8 °C for 30 min, and the resulting supernatants were quantified using the bicinchoninic acid assay according to the manufacturer’s instructions.

### 2.3. Protein Digestion

The protein digestion process was subjected to sequential processing as follows. A 100 μg quantity of protein from each sample was first diluted with triethylammonium bicarbonate buffer (TEAB, 100 mM final concentration), followed by reduction with tris (2-carboxyethyl) phosphine (10 mM final concentration) at 37 °C for 60 min. Alkylation was then performed with iodoacetamide (40 mM final concentration) in the dark at room temperature for 40 min. After centrifugation at 10,000× *g* for 20 min, the resulting precipitates were redissolved in 100 µL of 100 mM TEAB. Modified trypsin (Promega, Madison, WI, USA) was added at an enzyme-to-protein ratio of 1:50 (*w*/*w*), and the digestion was carried out overnight at 37 °C. The resulting peptides were vacuum-concentrated to dryness, reconstituted in 0.1% (*v*/*v*) trifluoroacetic acid, desalted on Oasis HLB cartridges, redried via vacuum centrifugation, and finally quantified by UV spectrophotometry using a NanoDrop ONE (Thermo Scientific, Waltham, MA, USA).

### 2.4. DIA Mass Spectrometry Analysis

The digested peptides were quantified by a VanquishNeo chromatographic system (Thermo Scientific, USA) coupled with an Orbitrap Astral mass spectrometer (Thermo Scientific, Waltham, MA, USA) at Majorbio Bio-Pharm Technology Co. Ltd. (Shanghai, China). Peptide separation was performed on a VanquishNeo system equipped with a Homemade column (15 cm × 100 μm, 1.7 μm). The column temperature was maintained at 40 °C, and the system backpressure was monitored within the range of approximately 400–600 bar. Mobile phase A consisted of water containing 2% acetonitrile and 0.1% formic acid, while mobile phase B comprised 80% acetonitrile and 0.1% formic acid in water. The gradient program was executed at a flow rate of 1 μL min^−1^ with the following program: 0–1 min, 8–17% B; 1–5.5 min, 17–55% B; 5.5–7 min, 55–99% B; 7–8 min, 99% B held. The total chromatographic run time was 8 min. Mass spectrometry data were acquired using Thermo Xcalibur software (version 4.7, Thermo Scientific, USA) operating in DIA positive-ion mode with an ion spray voltage of 2.2 kV. The mass spectrometry scanning range was 100–1700 *m*/*z*, with an isolation window width of 2 Th and a total of approximately 800 isolation windows.

### 2.5. Protein Identification and Bioinformatic Analysis

The DIA raw data were searched using Spectronaut software (Version 19, Biognosys AG, Schlieren, Switzerland). The protein database was constructed based on the genome of CN-3. The search parameters were set as follows: peptide length 7–52 amino acids, trypsin/P as the digestion enzyme, and a maximum of two missed cleavages allowed. Carbamidomethylation of cysteines was set as a fixed modification, and oxidation of methionine and protein N-terminal acetylation were set as variable modifications. Peptide identifications were filtered with a confidence threshold of ≥99%, and the extracted ion chromatogram width was restricted to ≤75 ppm. Both peptide and protein identifications were controlled at a 1% false discovery rate. Protein inference was performed using Spectronaut’s built-in IDPicker algorithm. Protein quantification was then carried out using the MaxLFQ algorithm, requiring a minimum of two unique peptides per protein. The “Proteotypic Filter” was enabled to restrict quantification to non-shared peptides at the protein level, thus avoiding potential biases arising from peptides shared among protein isoforms. The mass spectrometry proteomics data have been deposited in the ProteomeXchange Consortium (https://proteomecentral.proteomexchange.org (accessed on 14 August 2026)) via the iProX partner repository with the dataset identifier PXD082560. Functional annotation of all identified proteins was then performed primarily through automated database searches against the Clusters of Orthologous Groups of proteins (COG), Kyoto Encyclopedia of Genes and Genomes (KEGG), Gene Ontology (GO), Pfam, NR and Swiss-Prot databases, using the genome sequence of *D.* sp. CN-3 (NCBI accession number: JCCKJL000000000) as the reference. The annotations of key proteins were subsequently manually verified. These annotations are provided in the Supplementary Excel file (Supplementary Materials).

### 2.6. Heterologous Expression of alkB and CYP153 Genes

The activities of alkane hydroxylase were characterized by expressing the *alkB* or *CYP153* gene in *P. fluorescens* KOB2Δ1 and monitoring growth restoration on *n*-alkanes [30,32]. The *alkB* gene was amplified with the primers alkB-NdeI-F (5′-GGAATTCCATATGTCCAGCACCGAGTACATCAG-3′, 48.5% GC) and alkB-HindIII-R (5′-CCCAAGCTTTCACTTCACGGGCAGGAAGTC-3′, 56.7% GC). The *CYP153* gene was amplified with the primers CYP-NdeI-F (5′-GGGAATTCCATATGGTGAAGGTTTCCGATACGAT-3′, 44.1% GC) and CYP-HindIII-R (5′-CCCAAGCTTTCATGTGCCGGATTTCGGGGT-3′, 56.7% GC). All primers were designed using Primer Premier 5.0 software and verified for secondary structures and dimer formation using the software’s built-in analysis tools. The PCR program was as follows: 95 °C for 3 min; 30 cycles of 95 °C for 30 s, 58 °C for 30 s, and 72 °C for 90 s; a final extension at 72 °C for 10 min; and maintenance at 4 °C. The PCR product was ligated into pCom8 to yield the pCom8-alkB or pCom8-CYP plasmid, verified by sequencing, and electroporated into *P. fluorescens* KOB2Δ1. Transformants were selected on gentamicin (100 μg mL^−1^). Recombinants of KOB2Δ1 harboring pCom8 (negative control), pCom8-alkB, or pCom8-CYP were pre-cultured in LB medium, then harvested, washed three times with MSM, and transferred to 200 mL of MSM supplemented with 0.1% (*v*/*v*) of individual *n*-alkanes (C_12_, C_14_, C_16_, or C_28_). Cultures were incubated at 30 °C with shaking at 150 rpm for 38 d, and cell growth was monitored by measuring optical density at 600 nm. All the experiments were repeated in triplicate. Growth curves were further fitted to the modified Gompertz model [33] using GraphPad Prism (version 8.4.3, San Diego, CA, USA), yielding estimates of maximum OD (OD_max_), maximum specific growth rate (μ_max_), and lag phase duration (λ).

### 2.7. Statistical Analyses

Bioinformatics analysis was performed using the Majorbio Cloud platform (https://cloud.majorbio.com). Protein abundance values were normalized and retained on the original scale for statistical analysis. For fold-change-based identification of differentially expressed proteins (DEPs), only proteins quantified in all groups were included; proteins detected in a single group were excluded from this analysis and assessed independently. Statistical significance was evaluated using a two-tailed Student’s *t*-test, with *p*-values adjusted by the Benjamini–Hochberg procedure to control the false-positive rate from multiple testing. Proteins meeting the criteria of |log_2_FC| ≥ 1 and adjusted *p* < 0.05 were considered significantly changed. Growth curve data were analyzed using one-way ANOVA followed by Tukey’s post hoc test in GraphPad Prism, with statistical significance defined as *p* < 0.05.

## 3. Results and Discussion

### 3.1. Overview of DIA-Based Quantitative Proteomic Analysis

Strain CN-3 exhibits high degradation activity toward alkanes, with degradation rates of 97.2% for C_16_ and 75.3% for pristane, as previously reported in our earlier study [29] under the conditions described therein. This provides a solid foundation for interpreting the subsequent proteomic changes associated with alkane metabolism. The proteomic profiles of strain CN-3 grown on *n*-alkane (C_16_), branched alkane (pristane), and glucose (control) were systematically compared using DIA-based quantitative proteomics. A total of 2871 (glucose), 2894 (C_16_), and 2871 (pristane) proteins were identified, accounting for 82.12%, 82.78%, and 82.12% of the 3496 protein-coding genes predicted in the CN-3 genome, respectively. Venn diagram analysis further revealed 2854 proteins commonly identified across all three groups, with 0, 14, and 2 proteins uniquely identified in the glucose, C_16_, and pristane groups, respectively (Supplementary Materials, Figure S2). The high reproducibility of the proteomic data was confirmed by strong linear correlations observed among the four biological replicates within each treatment group (Supplementary Materials, Figure S3).

**Figure 1:**
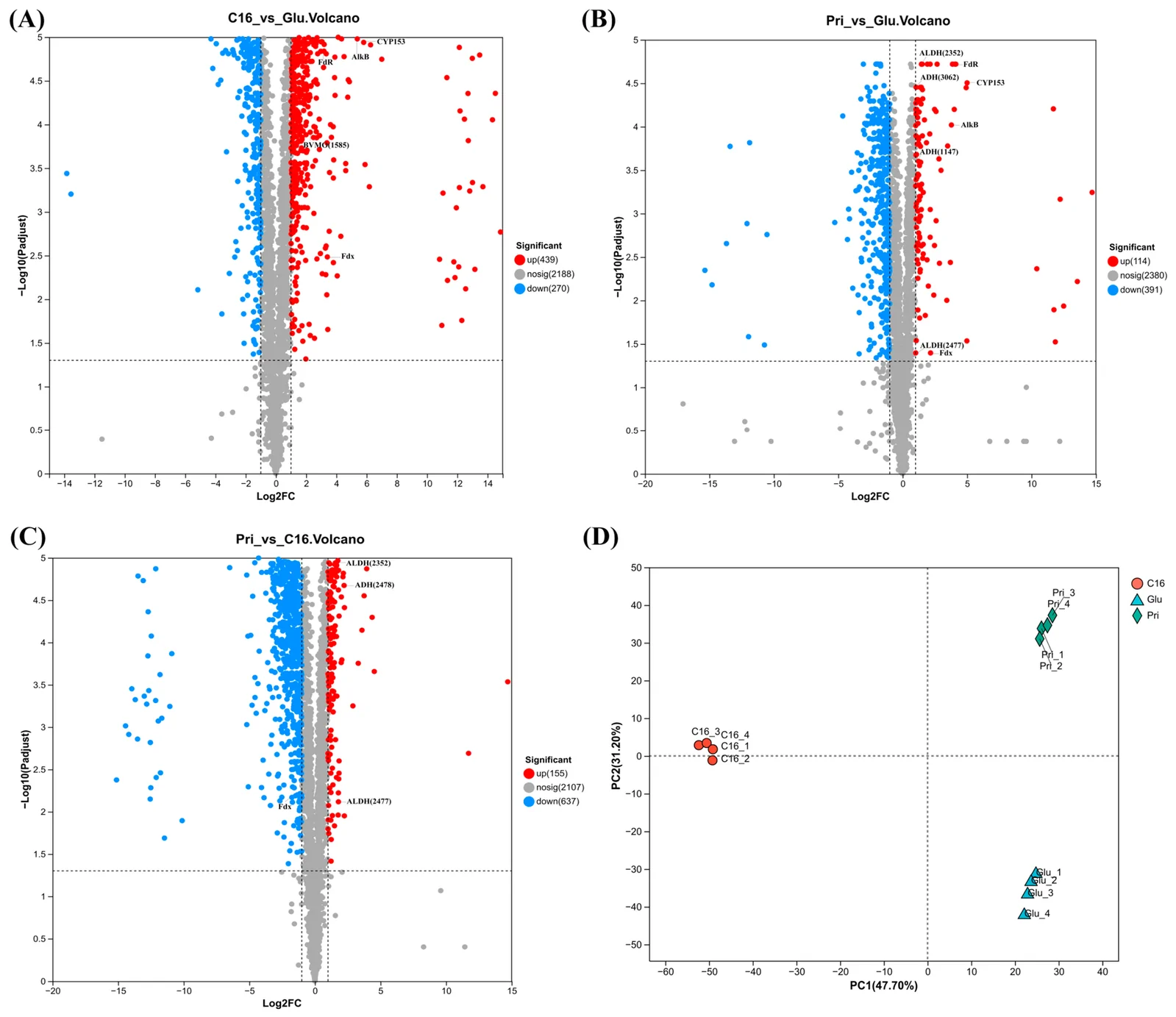
Proteomic profiles of strain CN-3 grown on C_16_, pristane (Pri), and glucose (Glu). (**A**–**C**) Volcano plots of DEPs for C_16_ vs. Glu (**A**), Pri vs. Glu (**B**), and Pri vs. C_16_ (**C**). Upregulated, downregulated, and non-significantly changed proteins are shown in red, blue, and gray, respectively; the thresholds were set at |log_2_FC| ≥ 1 and adjusted *p* < 0.05. Key proteins discussed in the text, including alkane hydroxylases (AlkB, CYP153), ferredoxin (Fdx), ferredoxin reductase (FdR), Baeyer–Villiger monooxygenases (BVMOs), alcohol dehydrogenases (ADHs), and aldehyde dehydrogenases (ALDHs), are labeled directly on the plot. (**D**) Principal component analysis (PCA) of replicate proteomes on growth of C_16_, pristane and glucose.

DEPs were identified using a fold change threshold of ≥2.0 (upregulated) or ≤0.5 (downregulated). A total of 709 DEPs were identified between the C_16_ and glucose groups, with 439 (61.92%) upregulated and 270 (38.08%) downregulated on C_16_ (Figure 1A). Additionally, 505 DEPs were detected between the pristane and glucose groups, of which 114 (22.57%) were upregulated and 391 (77.43%) were downregulated on pristane (Figure 1B). Furthermore, comparison between the two alkane substrates (pristane vs. C_16_) yielded 792 DEPs, with 155 (19.57%) upregulated on pristane (Figure 1C). Principal component analysis (PCA) further revealed a clear separation among the three treatment groups, whereas the biological replicates within each group clustered closely together (Figure 1D), corroborating the high reproducibility of the proteomic data. These findings reveal markedly different proteomic responses of strain CN-3 to C_16_ and pristane: the former elicited predominantly upregulated proteins consistent with active catabolic engagement, whereas the latter induced widespread downregulation, suggesting a stress-associated metabolic burden during branched-alkane utilization.

### 3.2. Functional Annotation and Pathway Enrichment Analyses

**Figure 2:**
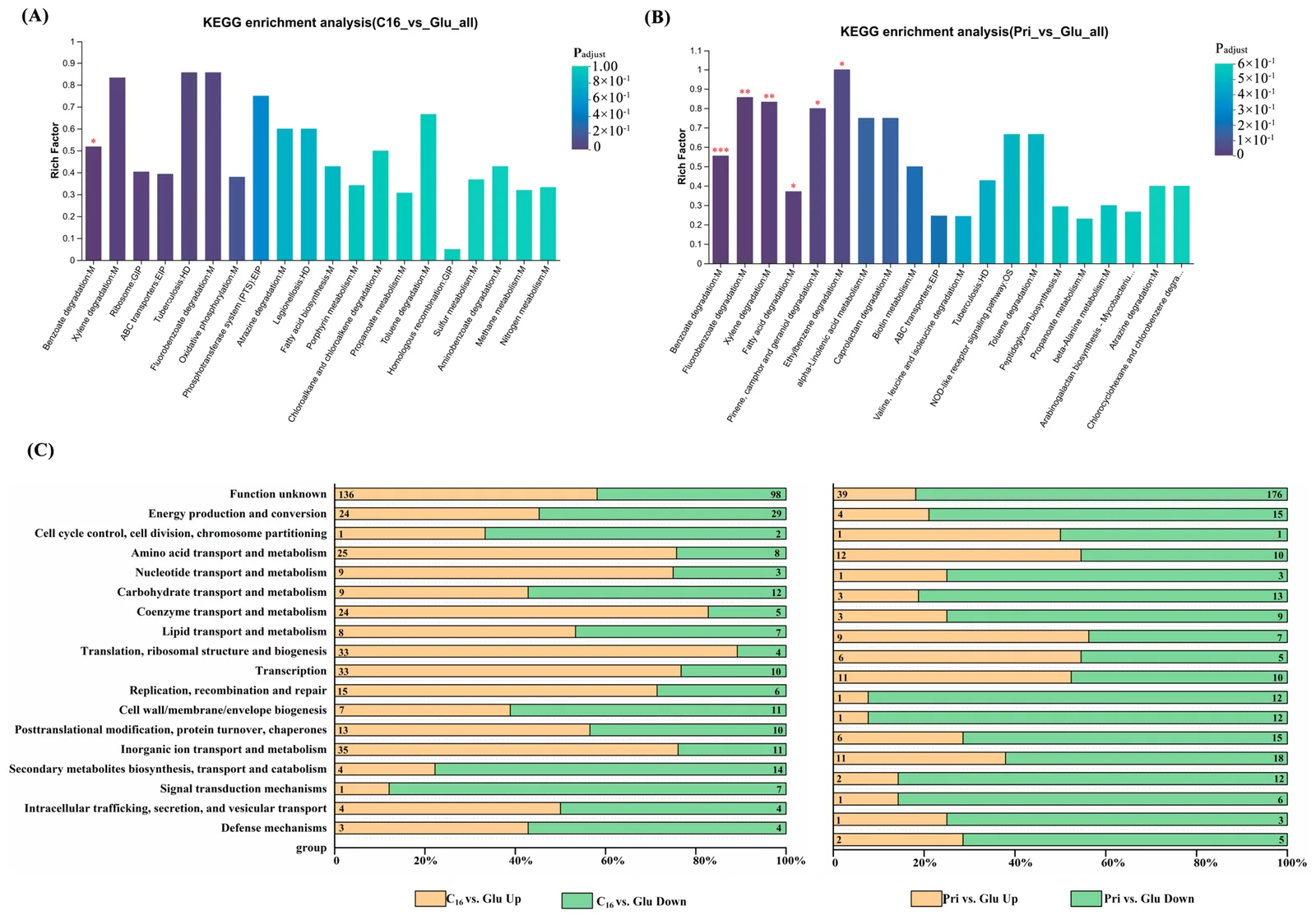
Functional annotation and pathway enrichment analysis of DEPs in *Dietzia* sp. CN-3. (**A**) KEGG pathway enrichment analysis of DEPs from C_16_ vs. Glu group. (**B**) KEGG pathway enrichment analysis of DEPs from Pri vs. Glu group. In both panels (**A**) and (**B**), the y-axis represents the enrichment factor, and the bar color indicates the adjusted *p* value. * indicates adjusted *p* < 0.05, ** indicates adjusted *p* < 0.01, and *** indicates adjusted *p* < 0.001. (**C**) COG-based functional classification of DEPs from C_16_ vs. Glu and Pri vs. Glu groups. The y-axis lists COG categories, and the x-axis indicates the percentage of DEPs per category. Upregulated (orange) and downregulated (green) genes are differentiated, with bar-embedded numbers representing the enriched DEP counts.

To further elucidate the differential proteomic expression profiles of the strain cultivated on C_16_, pristane and glucose, DEPs were subjected to KEGG pathway enrichment and COG functional classification analyses. KEGG enrichment analysis of the DEPs revealed distinct pathway profiles between the C_16_ and pristane conditions (Figure 2A,B). In the C_16_ versus glucose comparison, the most significantly enriched pathways included xylene degradation, fluorobenzoate degradation, benzoate degradation, ABC transporters, ribosome, and fatty acid biosynthesis (Figure 2A). Among these, xylene degradation (rich factor = 0.83, adjusted *p* = 0.066) and fluorobenzoate degradation (rich factor = 0.86, adjusted *p* = 0.089) exhibited the highest enrichment ratios, harboring 1 upregulated and 4 downregulated proteins, and 6 downregulated proteins, respectively (Supplementary Materials, Figure S4). The enrichment of multiple hydrocarbon catabolic pathways, together with fatty acid biosynthesis, indicates that C_16_ utilization activates both oxidative degradation routes and biosynthetic processes for carbon flux reallocation [17,34]. In the pristane versus glucose comparison, a broader range of KEGG pathways was significantly enriched compared with the C_16_ condition. Specifically, pathways involved in ethylbenzene degradation (rich factor = 1, adjusted *p* = 0.044), fluorobenzoate degradation (rich factor = 0.86, adjusted *p* = 0.002), xylene degradation (rich factor = 0.83, adjusted *p* = 0.009), pinene, camphor and geraniol degradation (rich factor = 0.8, adjusted *p* = 0.033), benzoate degradation (rich factor = 0.56, adjusted *p* = 0.0000447), and fatty acid degradation (rich factor = 0.37, adjusted *p* = 0.014) were prominently represented (Figure 2B). The broader enrichment of multiple oxygenase-associated pathways (e.g., xylene, ethylbenzene, and benzoate degradation) suggests that pristane degradation requires a more diverse enzymatic repertoire than C_16_ [7,9,29]. The significant enrichment of the ABC transporter pathway suggests that CN-3 enhances its capacity for the active uptake and intracellular translocation of hydrophobic alkanes.

Based on the COG database, the annotated DEPs from the C_16_ versus glucose and pristane versus glucose comparisons were assigned to 18 distinct functional categories (Figure 2C). In the categories of “Transcription”, “Translation, ribosomal structure and biogenesis”, “Amino acid transport and metabolism”, and “Lipid transport and metabolism”, the number of upregulated DEPs was higher than that of downregulated DEPs in both the C_16_ versus glucose and pristane versus glucose comparison groups. These functional categories were likely to enhance bacteria swiftly adapting to the petroleum hydrocarbon-polluted environments [17,34]. For the category of “Energy production and conversion”, downregulated DEPs outnumbered upregulated DEPs in both comparison groups, implying that cellular energy metabolism was consistently attenuated when cells were cultivated on alkanes compared with glucose, which was also consistent with our previous differential transcriptomic expression findings [29]. Annotation of these functional groups suggests that strain CN-3 exhibits metabolic features comparable to those of glucose-grown cells, irrespective of whether the alkane substrate is straight-chain (C_16_) or branched (pristane). In other functional categories such as “Nucleotide transport and metabolism”, “Coenzyme transport and metabolism”, “Inorganic ion transport and metabolism”, and “Post-translational modification, protein turnover, chaperones”, both the trend change and the number of DEPs varied considerably between the two comparison groups.

Together, functional annotation and pathway enrichment analyses revealed that, despite certain shared features, the strain exhibited distinct proteomic adaptation across multiple metabolic pathways between C_16_ and pristane conditions, indicating that CN-3 adopts substrate-specific metabolic strategies for straight-chain and branched-chain alkane degradation.

### 3.3. Alkane Uptake and Transport

Bacterial alkane uptake and transport are governed by multiple synergistic mechanisms [35,36]. For short-chain alkanes (<C_12_), which have relatively high aqueous solubility, passive diffusion across the cell membrane represents the primary entry route. For longer-chain alkanes, the hydrophobic outer membrane necessitates protein-mediated transport. It was reported that AlkL, FadL, AupA, and OmpT have been identified as key proteins of this process in Gram-negative bacteria [35]. Furthermore, cells also enhance alkane uptake through biosurfactant-mediated solubilization of hydrocarbons, modulating cell surface hydrophobicity (CSH) to increase cell-hydrocarbon adhesion, and forming biofilms on alkane droplets to promote direct interfacial contact [19,37]. Among the characterized transport systems, two major outer membrane protein families, AlkL/OmpW and FadL families, have been implicated as channels facilitating the transmembrane passage of alkanes in Gram-negative bacteria [35,37]. In addition, some bacteria, such as *Marinobacter hydrocarbonoclasticus*, employ specialized AupA/B to acquire alkanes from surfactant-solubilized micelles. In the obligate hydrocarbonoclastic bacterium *A. borkumensis* SK2, the outer membrane lipoprotein gene *blc* has been implicated in alkane uptake and transport [38]. No homologs of OmpW, AlkL, FadL or AupA/B were detected in strain CN-3. Instead, a membrane-anchored lipoprotein (Die3_GM000935) and a zinc ABC transporter substrate-binding protein (Die3_GM000578)—the latter mediating active solute transport across the cytoplasmic membrane in Gram-positive bacteria—were upregulated under C_16_ conditions relative to glucose (Table 2). Glycolipids are typical biosurfactants produced by hydrocarbon-degrading bacteria [37,39]. Herein, we identified three putative glycosyltransferases involved in glycolipid biosynthesis. Among these, the glycosyltransferase (Die3_GM003051) showed significant upregulation both in C_16_ relative to glucose and in pristane.

In addition, under alkane stress, bacterial cells appear to adopt an adaptive strategy by enhancing poly-L-glutamine (PLG) biosynthesis, thereby increasing CSH and promoting adhesion to alkane droplets [40,41,42,43]. In support of this notion, the glutamate synthase (Die3_GM000255) and glutamine synthetase (Die3_GM002030) were both upregulated under C_16_ relative to glucose conditions. Consistent with this expression pattern, strain CN-3 exhibited a high CSH of 76.51 ± 4.34% when cultivated on C_16_, whereas a substantially lower CSH (36.5 ± 4.64%) was observed under pristane conditions [29].

### 3.4. Terminal and Subterminal Oxidation of n-Alkane

**Figure 3:**
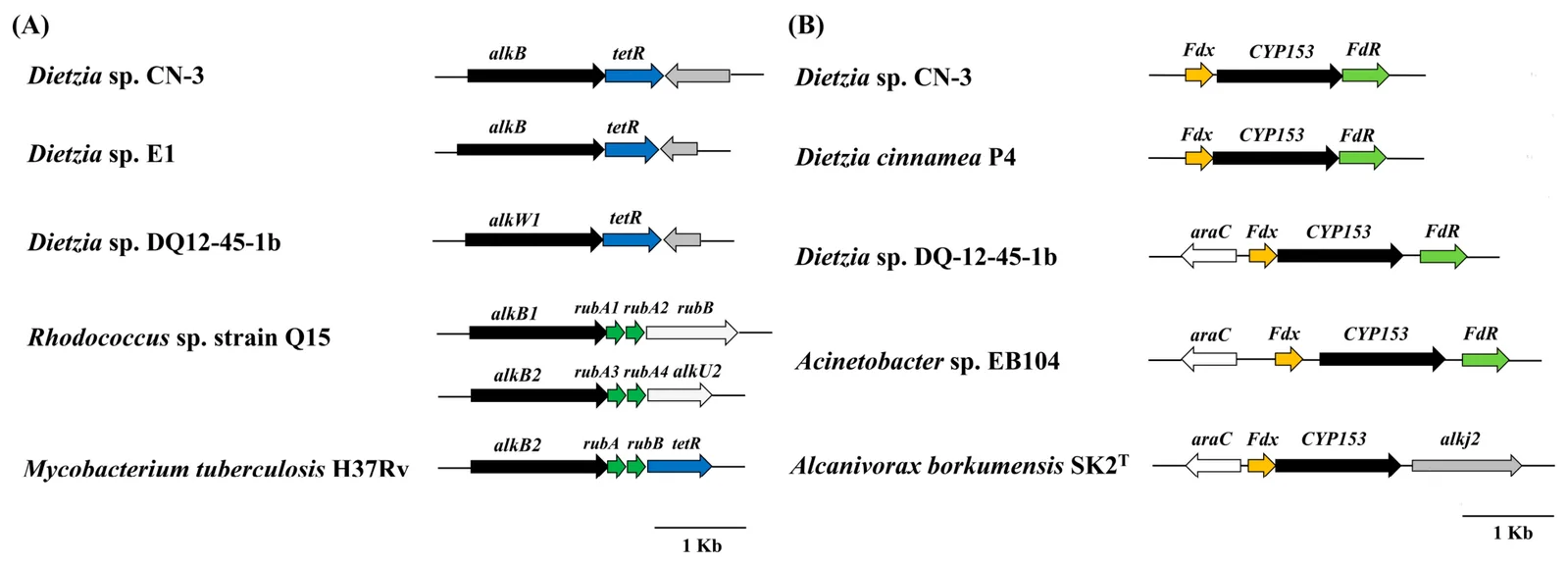
Gene organization of *alkB* and *CYP153* genes in different alkane-degrading bacteria. (**A**) *alkB*, alkane hydroxylase gene; *rubA*, rubredoxin gene; *rubB*, rubredoxin reductase gene; *tetR/alkU2,* TetR family transcriptional regulator gene; (**B**) *CYP153*, cytochrome P450 hydroxylase gene; *Fdx*, ferredoxin gene; *FdR*, ferredoxin reductase gene; *araC*, AraC family transcriptional regulator gene; *alkj2,* alcohol dehydrogenase gene.

As the initial and rate-determining step in the multi-step oxidative degradation of alkanes, hydroxylation is catalyzed by monooxygenases. These enzymes consume molecular oxygen and generate reactive oxygen species, which are required to overcome the thermodynamic stability of the alkane C-H bond [2,44]. In our proteomic analysis, we observed that several alkane monooxygenases exhibited significant changes in abundance during growth on C_16_ compared with glucose (Supplementary Materials, Table S1), reflecting the activation of the terminal and subterminal oxidation pathway. Although the AlkB system traditionally requires three separate components (AlkB, rubredoxin, and rubredoxin reductase) for alkane hydroxylation, one alkane hydroxylase–rubredoxin fusion protein AlkB (Die3_GM000017) was identified as being significantly upregulated (41.77-fold) in the C_16_ versus glucose group. Notably, similar AlkB–rubredoxin fusion proteins have recently been reported in several Gram-positive alkane-degrading bacteria, including *Dietzia* sp. E1, *Nocardioides* sp. CF8, and *D.* sp. DQ12-45-1b [30,45]. Nevertheless, the functional advantage for alkane hydroxylation remains unclear. The *alkB* gene encodes a 495-amino-acid protein that shares 89.66% and 85.63% sequence identity with the alkane hydroxylase of *D.* sp. E1 and *D.* sp. DQ12-45-1b, respectively. The *alkB* gene organization is highly conserved across the genus *Dietzia*, characterized by a TetR family transcriptional regulator located immediately downstream of *alkB*. This arrangement differs markedly from that observed in *Rhodococcus* sp. strain Q15 and *Mycobacterium tuberculosis* H37Rv, where the rubredoxin gene *rubA* and rubredoxin reductase *rubB* are two separate genes, rather than being fused or absent (Figure 3A). In *D.* sp. DQ12-45-1b, the AlkB-fused rubredoxin domain forms a novel phylogenetic cluster with those from Gram-positive fusion gene clusters, distinct from AlkG1/AlkG2. The encoded AlkW1 hydroxylase requires its fused rubredoxin for activity and supports growth on *n*-alkanes up to C_32_. In the Gram-negative alkane-degrading bacterium *Fontimonas thermophila*, the cryo-electron microscopy structure of the AlkB–rubredoxin fusion complex (FtAlkBG) reveals that the rubredoxin domain binds to the diiron center of AlkB in a conformation conducive to direct electron transfer, while substrate binding allosterically enhances their interaction, thereby synergistically promoting catalytic efficiency [46,47]. In our dataset, the AlkB–rubredoxin fusion was significantly upregulated during growth on C_16_ compared with glucose, suggesting a potential role in alkane catabolism. Similar fusion proteins in related *Dietzia* strains have been shown to support growth on *n*-alkanes, although the precise functional advantage and electron-transfer efficiency of this fusion in CN-3 require direct experimental validation.

The cytochrome P450 hydroxylase CYP153 (Die3_GM000105), which exhibits 85.19% amino acid sequence identity to the P450 alkane hydroxylase of *N.* sp. CF8, showed a significant 32.02-fold upregulation in the C_16_ versus glucose group. The CYP153 hydroxylase is part of a putative gene cluster that also includes genes encoding a ferredoxin (Fdx, Die3_GM000104) and a ferredoxin reductase (FdR, Die3_GM000106). The FdR accepts electrons from NAD(P)H and reduces Fdx, which subsequently serves as the direct electron donor to the terminal CYP153 hydroxylase [9,24]. In response to growth on C_16_ relative to glucose, the Fdx and FdR were significantly upregulated 10.58- and 9.05-fold, respectively. The gene organization of CYP153 is very similar to that of *D. cinnamea* P4 and DQ-12-45-1b, whereas in strain SK2, the gene immediately downstream of CYP153 is the alcohol dehydrogenase gene *alkJ2* (Figure 3B). In *A. borkumensis* SK2, two CYP153 cytochromes (ABO_0201, three-fold; ABO_2288, two-fold) were significantly upregulated during growth on *n*-C_14_ compared to pyruvate, implicating their involvement in terminal alkane oxidation [9]. Likewise, in *Tsukamurella tyrosinosolvens* PS2, genes encoding a cytochrome P450 monooxygenase, ferredoxin VI, and the putida redoxin reductase were all upregulated on hexadecane relative to sucrose, further supporting the involvement of CYP153-type systems in alkane oxidation [48]. A transposase gene (Die3_GM000100) was found adjacent to the CYP153 gene cluster and was significantly induced (2.49-fold) in the C_16_ versus glucose treatment, consistent with its potential role in genomic plasticity.

**Figure 4:**
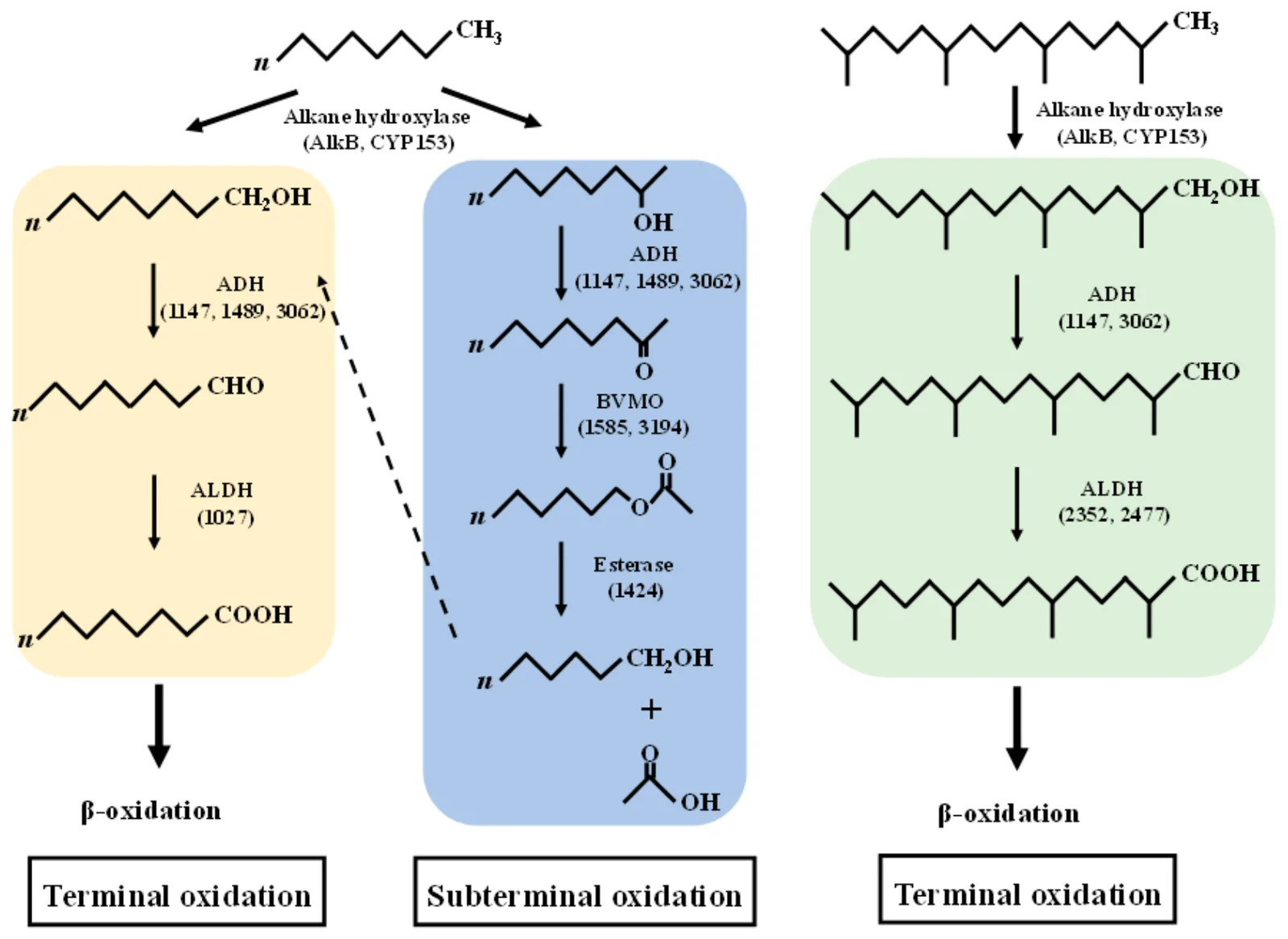
Proposed terminal and subterminal oxidation pathways for C_16_ and pristane in *Dietzia* sp. CN-3. The overall pathway is presented as a working model derived from proteomic data. Direct verification of individual intermediate conversions will require future metabolomic and enzymatic studies. ADH, alcohol dehydrogenase; ALDH, aldehyde dehydrogenase; BVMO, Baeyer–Villiger monooxygenase. Numbers in parentheses are the last four digits of the key enzyme IDs.

In addition to the terminal oxidation mediated by AlkB and CYP153 systems, subterminal oxidation of C_16_ has been documented as an alternative metabolic pathway in the CN-3 strain. The proposed degradation pathway is illustrated in Figure 4. This pathway proceeds via a secondary alcohol intermediate, which is dehydrogenated to the corresponding ketone. The ketone is converted to an ester by a Baeyer–Villiger monooxygenase (BVMO), and the ester is subsequently hydrolyzed into a primary alcohol and a fatty acid by an esterase. In this study, two Baeyer–Villiger monooxygenases (Die3_GM001585 and Die3_GM003194) were identified in our proteomics data and showed 4.82- and 2.74-fold greater expression, respectively, in response to C_16_ relative to glucose. Multiple sequence alignment of different BVMOs indicated that these two BVMOs possess the characteristic Type I BVMO fingerprint sequence (FXGXXXHXXXW) [49]. They also contain two Rossmann fold motifs (GXGXX) for cofactor binding, and the motif (GGXWXXXXYPGXXXD) [50] that distinguishes BVMOs from other flavin monooxygenases (Supplementary Materials, Figure S5). Likewise, an esterase (Die3_GM001424) was also induced (2.07-fold), suggesting its potential involvement in the subsequent ester hydrolysis step of the subterminal oxidation pathway. Particularly, these two BVMOs and one esterase were only induced by C_16_ relative to glucose, but remained uninduced by pristane, indicating a substrate-specific response. Through physiological, biochemical, and bioinformatics analyses, AlmA from *Acinetobacter baylyi* ADP1 was identified as a Baeyer–Villiger monooxygenase that catalyzes the conversion of aliphatic 2-ketones (C_10_–C_16_) to their corresponding esters [18]. The subterminal pathway offers greater carbon efficiency for converting long-chain alkanes to short-chain alcohols, which may explain why certain bacteria adopt this route to thrive on complex petroleum hydrocarbon substrates.

In both terminal and subterminal alkane oxidation pathways, alcohol dehydrogenases (ADHs) and aldehyde dehydrogenases (ALDHs) play critical roles, catalyzing the sequential oxidation of alcohols to their corresponding aldehydes and further to fatty acids [2,46]. Three alcohol dehydrogenases (Die3_GM001147, Die3_GM001489 and Die3_GM003062) and one aldehyde dehydrogenase (Die3_GM001027) were significantly differentially expressed on *n*-C_16_. In summary, the coexistence of terminal and subterminal oxidation routes in alkane-degrading bacteria reflects a high degree of metabolic flexibility. However, the proposed pathways should be explicitly framed as hypothetical models that require further verification. They are predicted to rely on distinct enzymes (e.g., AlkB/CYP153 hydroxylases, alcohol/aldehyde dehydrogenases, Baeyer–Villiger monooxygenases, and esterases) that collectively convert *n*-alkanes to acyl-CoA derivatives. Although our current dataset primarily reflects the cellular capacity for alkane degradation, it nonetheless provides a valuable foundation for further mechanistic exploration. To fully delineate the functional operation of these pathways and to quantify the actual intermediates, future work should integrate targeted metabolomics, in vitro enzymatic assays, and kinetic analyses.

### 3.5. Terminal Oxidation of the Branched Alkane

Branched alkanes are more recalcitrant to microbial degradation than *n*-alkanes due to their greater persistence, toxicity, and bioaccumulation potential, making them useful indicators of petroleum degradation in contaminated environments [51,52]. This recalcitrance is primarily attributed to the methyl branches along the carbon skeleton, which hinder the uptake of the hydrocarbons into the cell and impede β-oxidation at the branch points [10,53]. In our proteomic analysis, several alkane monooxygenases showed significant abundance changes in pristane versus glucose groups, indicating the induction of terminal oxidation machinery for branched-chain alkane catabolism. Specifically, the alkane hydroxylase–rubredoxin fusion AlkB (Die3_GM000017) and the cytochrome P450 hydroxylase CYP153 (Die3_GM000105) were exclusively upregulated by 13.77- and 32.02-fold, respectively. This proteomic profile aligns with our previous comparative transcriptomics and RT-qPCR data, which showed that CYP153 was strongly induced by both C_16_ and pristane, whereas *alkB* was moderately induced by C_16_ but only weakly responsive to pristane [29]. Importantly, the dominant contribution of CYP153 over AlkB in our study differs from that reported in *D.* sp. DQ12-45-1b, where AlkB played a more prominent role in alkane oxidation [17,24], highlighting the strain-specific strategies for alkane hydroxylation. In *A. hongdengensis* A-11-3, pristane induces P450-3 transcription [22], whereas in *A. borkumensis* SK2, P450-3 is markedly upregulated on pristane relative to *n*-alkanes or pyruvate [54]; proteomics further implicates one AlmA and three CYP153 P450s in pristane oxidation [9]. In addition, sequential oxidation reactions are commonly catalyzed by cytochrome P450 enzymes, particularly in the context of xenobiotic metabolism [55]. The ferredoxin (FdX, Die3_GM000104) and the ferredoxin reductase (FdR, Die3_GM000106) were also significantly induced by 3.14- and 17.45-fold, respectively. In summary, these four DEPs are candidate catalysts for the initial hydroxylation of pristane to pristanol. 

The subsequent oxidation of pristanol to pristyl aldehyde is primarily mediated by alcohol dehydrogenases (ADHs), alongside alcohol oxidases (AOXs) as a secondary pathway. Alcohol dehydrogenases are nicotinamide-dependent, while alcohol oxidases are flavin-dependent and generate hydrogen peroxide [53,56]. In our proteomic dataset, two ADH homologs (Die3_GM001147, 2.35-fold; Die3_GM003062, 2.07-fold) were significantly induced during growth on pristane, whereas no AOX homolog was detected. For the oxidation of pristyl aldehyde to pristanic acid, two aldehyde dehydrogenases (Die3_GM002352 and Die3_GM002477) exhibited significant upregulation under pristane-amended conditions. Together, these enzymes delineate a putative terminal oxidation pathway for pristane, and to our knowledge, this study provides preliminary proteomic evidence for such a route in *Dietzia*. Similarly, the proposed terminal oxidation pathway for pristane warrants further validation through complementary approaches, including targeted metabolomics and in vitro enzymatic characterization, to confirm the predicted intermediates and to assess the actual route.

### 3.6. Fatty Acid β-Oxidation

**Figure 5:**
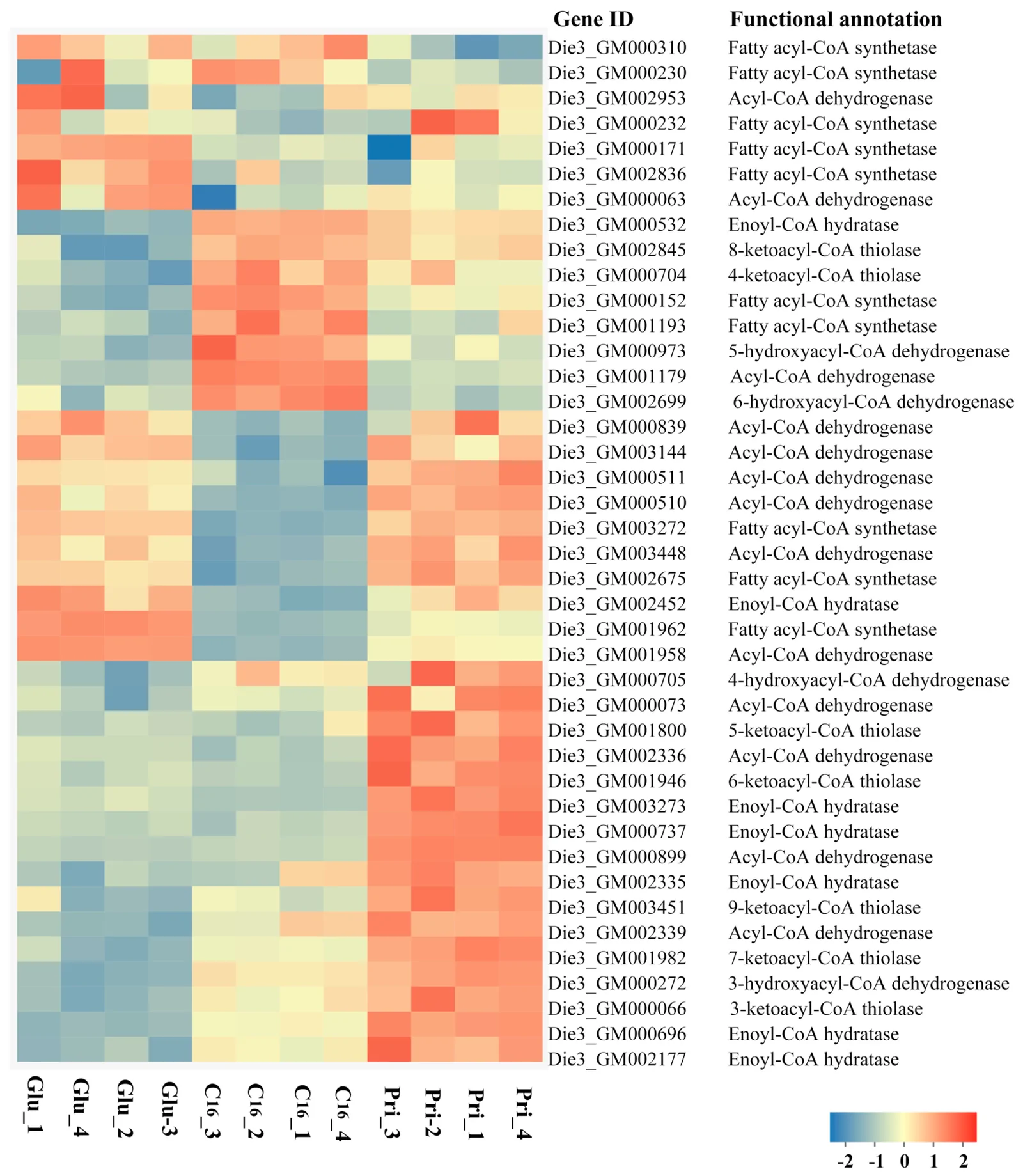
Heatmap showing the abundance profiles of representative proteins associated with fatty acid β-oxidation in CN-3 strain grown on C_16_, pristane (Pri), or glucose (*n* = 4 biological replicates per condition). Hierarchical clustering was performed using Euclidean distance as the distance metric and complete-linkage as the agglomeration method for heatmap construction. All proteins displayed in this heatmap were preselected based on the differential-expression criteria (fold change ≥ 1 and adjusted *p* < 0.05). Red and blue colors represent relatively higher and lower abundance, respectively, across the three culture conditions.

The oxidation of alkanes generates large amounts of fatty acids, which are subsequently metabolized by the β-oxidation pathway [57,58,59]. The expression patterns of the identified β-oxidation proteins indicate that they are differentially implicated in the catabolism of the *n*-alkane (C_16_) and the branched-chain alkane (pristane) (Figure 5). Prior to metabolic utilization, a fatty acid must be activated to its acyl-CoA derivative in an ATP-consuming reaction, which is catalyzed by fatty acyl-CoA synthetase (FadD, also known as long-chain-fatty-acid-CoA ligase) and constitutes the rate-limiting step of β-oxidation [57]. Among the 10 FadD homologs identified in the C_16_ versus glucose comparison, three were induced and seven were repressed. In contrast, the pristane versus glucose comparison revealed a more balanced response, with five homologs upregulated and five downregulated. Acyl-CoA dehydrogenase (FadE) catalyzes the α, β-dehydrogenation of acyl-CoA to trans-Δ^2^-enoyl-CoA, with FAD as the electron acceptor and being reduced to FADH_2_; this reaction constitutes the first dehydrogenation step of β-oxidation. Three FadEs showed significant upregulation during growth on pristane, including Die3_GM000073 (3.38-fold), Die3_GM000899 (16.07-fold) and Die3_GM002339 (2.64-fold), while only one FadE (Die3_GM001179, 3.13-fold) was significantly upregulated on C_16_. Furthermore, enoyl-CoA hydratase (FadH) catalyzes the hydration of trans-Δ^2^-enoyl-CoA to produce 3-hydroxyacyl-CoA via the stereospecific addition of a water molecule across the trans double bond. This step was supported by two upregulated FadH homologs (Die3_GM000696, 3.23-fold; Die3_GM003273, 3.03-fold) on pristane, compared with only one (Die3_GM000532, 2.28-fold) on C_16_. The second dehydrogenation of the cycle is executed by 3-hydroxyacyl-CoA dehydrogenase (FadB), which oxidizes the C-3 hydroxyl group of 3-hydroxyacyl-CoA to a ketone, yielding β-ketoacyl-CoA. One FadB homolog (Die3_GM002699, 3.43-fold) was significantly upregulated under C_16_ versus glucose conditions, and a distinct one (Die3_GM000272, 2.39-fold) under pristane conditions, exhibiting different expression patterns during hydrocarbon utilization. This is consistent with the known substrate specificity variations among FadB paralogs and the frequent occurrence of multiple operons in bacterial genomes [57,58]. Ultimately, 3-ketoacyl-CoA thiolase (FadA) mediates the thiolysis of 3-ketoacyl-CoA in the final β-oxidation step, releasing acetyl-CoA and yielding an acyl-CoA shortened by two carbons. Three FadA homologs were significantly upregulated on pristane, but none on C_16_.

In Gram-negative bacteria of *Acinetobacter oleivorans* DR1, β-oxidation genes *fadD*, *fadE*, *fadB*, and *fadH* were induced in the C_16_ versus succinate comparison [59]. Shotgun proteomics further revealed that *A. borkumensis* SK2 deploys distinct β-oxidation enzyme sets for fatty acid catabolism, with substrate-specific expression profiles observed between C_16_ and pristane [9]. In our proteomics analysis, the number of upregulated proteins involved in β-oxidation was considerably higher under pristane-amended conditions than under C_16_-amended conditions, suggesting that the degradation of the branched-chain alkane may require a broader enzymatic repertoire. Unlike straight-chain C_16_, which is processed via core β-oxidation, pristane necessitates auxiliary enzymes to bypass branch-induced steric hindrance, which may account for the more extensive protein response observed [60].

### 3.7. Functional Complementation of alkB and CYP153 Genes in P. fluorescens KOB2Δ1

**Figure 6:**
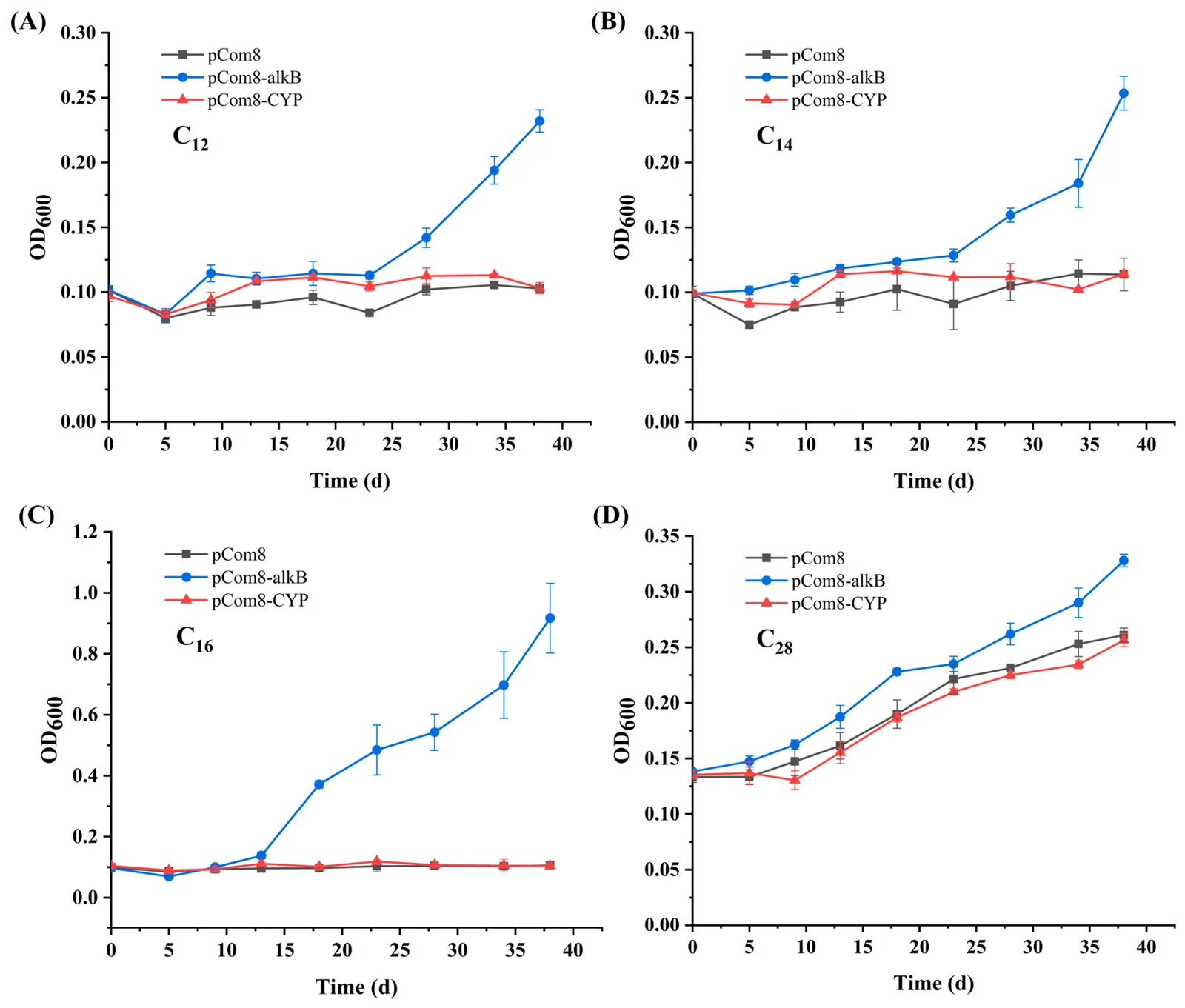
Growth of *P. fluorescens* KOB2Δ1 recombinants harboring pCom8 (control), *alkB*, or *CYP153* in mineral salts medium supplemented with *n*-alkanes C_12_ (**A**), C_14_ (**B**), C_16_ (**C**), or C_28_ (**D**) as the sole carbon source. Growth was assessed by measuring OD_600_ over the incubation period in three biological replicates.

To characterize the alkane hydroxylation activities of strain CN-3, we employed the *alkB*-deficient host *P. fluorescens* KOB2Δ1 for heterologous expression of its *alkB* and CYP153 genes. *P. fluorescens* KOB2Δ1 has been reported to be unable to grow on C_12_–C_16_ *n*-alkanes, while retaining the ability to utilize C_18_–C_28_ *n*-alkanes [30,31,32]. To evaluate their substrate specificity, we examined the growth of KOB2Δ1 recombinants expressing *alkB* or *CYP153* on *n*-alkanes of varying chain lengths (C_12_–C_28_, Figure 6). On C_12_, the pCom8-alkB strain achieved a maximum specific growth rate (μ_max_) of 0.079 d^−1^, significantly outperforming both the pCom8 control and the CYP153 strain (*p* < 0.05), with a lag phase of approximately 26.8 d. Similar growth kinetics were observed on C_14_ (μ_max_ = 0.064 d^−1^), indicating comparable substrate utilization. On C_16_, the pCom8-alkB strain exhibited improved growth, entering exponential phase earlier (λ ≈ 14.0 d) and reaching a substantially higher maximal OD_600_ (0.913) than observed on C_12_ and C_14_. Although the host strain KOB2Δ1 inherently utilized C_28_ (μ_max_ = 0.052 d^−1^), the pCom8-alkB recombinant exhibited a significantly faster growth rate (μ_max_ = 0.065 d^−1^, *p* < 0.05) and higher final biomass (OD_max_ = 0.333, *p* < 0.05) relative to the pCom8 control. These quantitative analyses demonstrate that *alkB* confers growth on C_12_–C_16_ and enhances the endogenous activity on C_28_. Similarly, in the *D.* sp. DQ12-45-1b, the *alkW1* recombinant regained the capacity to grow on C_18_–C_32_ *n*-alkanes and degraded a wider range of these substrates compared with the alkW1ΔRd mutant [61]. Likewise, in *Rhodococcus* sp. strain CH91, *alkB1* and *alkB2* showed distinct substrate ranges: KOB2Δ1 (pCom8/CH91alkB1) recombinant enhanced degradation of C_16_–C_26_ *n*-alkanes, whereas KOB2Δ1 (pCom8/CH91alkB2) recombinant extended the profile to C_16_–C_30_, relative to the vector control [31].

In contrast, the CYP153-expressing strain showed no significant growth advantage over the control on any substrate tested (*p* > 0.05 for all comparisons). Two possible explanations may account for this observation. First, the heterologously expressed *CYP153* gene may not be efficiently expressed, properly folded, or enzymatically active in the host strain. Second, the absence or insufficient supply of compatible electron transfer partners (ferredoxin and ferredoxin reductase), which were not independently examined in this study, may preclude the functional coupling required for CYP153-mediated hydroxylation. We attempted to co-express *CYP153* together with its cognate ferredoxin and ferredoxin reductase in this study; however, we were unable to obtain a stable co-expression construct in the pCom8 vector despite multiple cloning attempts. Consequently, the expression of ferredoxin and ferredoxin reductase was not examined independently of CYP153, and no strain co-expressing all three components was available for expression or activity analysis. This limitation prevents us from determining whether the absence of a growth advantage is caused by insufficient CYP153 expression, folding, or activity, or by the lack of compatible redox partners. Notably, a functional P450 hydroxylase from *A. dieselolei* B-5 has been validated in the same KOB2Δ1 background using a different vector (pCom12) [62], confirming the host’s capacity to support P450 activity when redox partners are properly supplied, although the pCom12 architecture differs from that of our pCom8 system. Therefore, the function of the CYP153 gene remains to be fully elucidated. Future efforts should focus on optimizing expression conditions, particularly by co-expressing cognate ferredoxin and ferredoxin reductase in a compatible vector system and independently verifying the expression of all components at the mRNA and protein levels, or by testing alternative host systems.

## 4. Conclusions

In conclusion, this study was designed to systematically compare the substrate-specific proteomic responses of strain CN-3 to linear (*n*-hexadecane) and branched (pristane) alkanes. Our DIA-based quantitative proteomics approach identified differential expression of key enzymes potentially involved in alkane uptake, terminal/subterminal oxidation, and fatty acid β-oxidation. Functional validation of AlkB by heterologous expression further supports its role in the utilization of medium- to long-chain alkanes, although the overall pathway remains a working model inferred from proteomic data and requires future confirmation by metabolomics. Importantly, these protein-level data point to a post-transcriptional regulatory layer in *Dietzia* metabolism, as enzyme abundances were not proportional to their transcript levels—highlighting the unique value of proteomics in revealing regulatory complexity beyond genomic and transcriptional profiling.

While the substrate-specific metabolic strategies may contribute to the adaptability of CN-3 in hydrocarbon-contaminated environments, direct ecological competition experiments would be required to substantiate any fitness advantage in situ. In particular, the proposed terminal and subterminal oxidation routes, together with the metabolic strategies, should be regarded as working hypotheses requiring experimental validation through targeted metabolomics, in vitro enzymatic assays, and kinetic analyses. Our findings establish a foundational proteomic framework for the genus *Dietzia*, providing a valuable resource and generating testable hypotheses regarding its regulatory and metabolic adaptation mechanisms.

## Figures and Tables

**Table 1:** Bacterial strains and plasmids used in this study.

Strain or Plasmid	Relevant Phenotype, Genotype, or Characteristics	Source or Reference
Strains		
*Pseudomonas fluorescens* KOB2Δ1	*alkB* knockout strain of *P. fluorescens* CHA0	[30]
*D*. sp. CN-3	Grows on C_12_–C_36_ *n*-alkanes, branched alkanes and crude oil	[8]
*Escherichia coli* DH5α	Cloning strain	Vazyme
Plasmids		
pCom8	Broad-host-range expression vector with P_alkB_, Gm^r^*, oriT*, *alkS*	[32]
pCom8-alkB	pCom8 with *D.* sp. CN-3 *alkB* gene, Gen^r^	This study
pCom8-CYP	pCom8 with *D.* sp. CN-3 *CYP153* gene, Gen^r^	This study

**Table 2:** Differential expression proteins related to alkane uptake and transport.

Pathways	Gene ID	Protein Annotation	C_16_/Glu	Pri/Glu	Pri/C_16_
Biosynthesis of outer membrane lipoprotein	Die3_GM000578	Zinc ABC transporter substrate-binding protein	2.02	−0.22	−2.24
	Die3_GM000935	Lipoprotein	0.70	−0.67	−1.37
Biosynthesis of glycolipid	Die3_GM002152	Glycosyltransferase	3.30	−10.22	−13.52
	Die3_GM002374	Glycosyltransferase	1.61	−0.01	−1.62
	Die3_GM003051	Glycosyltransferase	10.83	6.76	−4.07
Biosynthesis of PLG layer	Die3_GM000255	Glutamate synthase (NADPH) small chain GltD	1.44	0.49	−0.95
	Die3_GM000431	Glutamine synthetase family protein	−0.58	0.48	1.06
	Die3_GM002030	Glutamine synthetase	1.13	−0.32	−1.45

Data are presented as log_2_FC for C_16_/glucose, pristane/glucose, and pristane/C_16_. Downregulated genes are marked with “−” values. All proteins listed in this table met the statistical significance criteria (adjusted *p* < 0.05).

## Data Availability

The original contributions presented in this study are included in the article and Supplementary Materials. Further inquiries can be directed to the corresponding author.

## Supplementary Materials

The following supporting information can be downloaded at: https://www.mdpi.com/article/10.3390/microorganisms14092100/s1. Figure S1. Schematic workflow of the Data-Independent Acquisition (DIA) project. Figure S2. Venn diagram showing the overlap of identified proteins among the C_16_, pristane (Pri) and glucose (Glu) treatments. Figure S3. Correlation analysis of samples from the strain CN-3 under pristane (Pri), glucose (Glu), and C_16_ treatments. Biological replicates (1–4) are indicated for each group. Figure S4. KEGG enrichment chord diagrams of DEPs in strain CN-3 for the treatment groups C_16_ vs Glu (A) and Pri vs Glu (B). Figure S5. Multiple sequence alignment of different Baeyer-Villiger monoxygenases (BVMOs). Table S1. Differentially expressed proteins related to oxidation of C_16_ and pristane.
