# Supplementary material for: Quantitative Proteomics Reveals Substrate-Specific Metabolic Adaptations for n-Alkane and Branched Alkane Degradation in Dietzia sp. CN-3
Source: Microorganisms. 2026 Sep 19;14(9):2100. doi: 10.3390/microorganisms14092100 (PMC13609444; doi:10.3390/microorganisms14092100)
Supplement: Supplementary file 1 [file microorganisms-14-02100-s001.zip › microorganisms-4525226-supplementary.pdf]

microorganisms

Article

Quantitative Proteomics Reveals Substrate-Specific Metabolic Adaptations for *n*-Alkane and Branched Alkane Degradation in *Dietzia* sp. CN-3

Weiwei Chen [1,†], Xin Zhang [1,†], Nian Liu [1], Jun Min [2], Shiwei Cheng [1,*]

[1] School of Life Sciences, Ludong University, Yantai 264025, China; wwchen@ldu.edu.cn (W.C.); 15192081793@163.com (X.Z.); LNian0426@163.com (N.L.)
[2] Yantai Institute of Coastal Zone Research, Chinese Academy of Sciences, Yantai 264003, China; jmin@yic.ac.cn
* Correspondence: swcheng@ldu.edu.cn
† These authors contributed equally to this work.

 https://doi.org/10.3390/microorganisms14092100

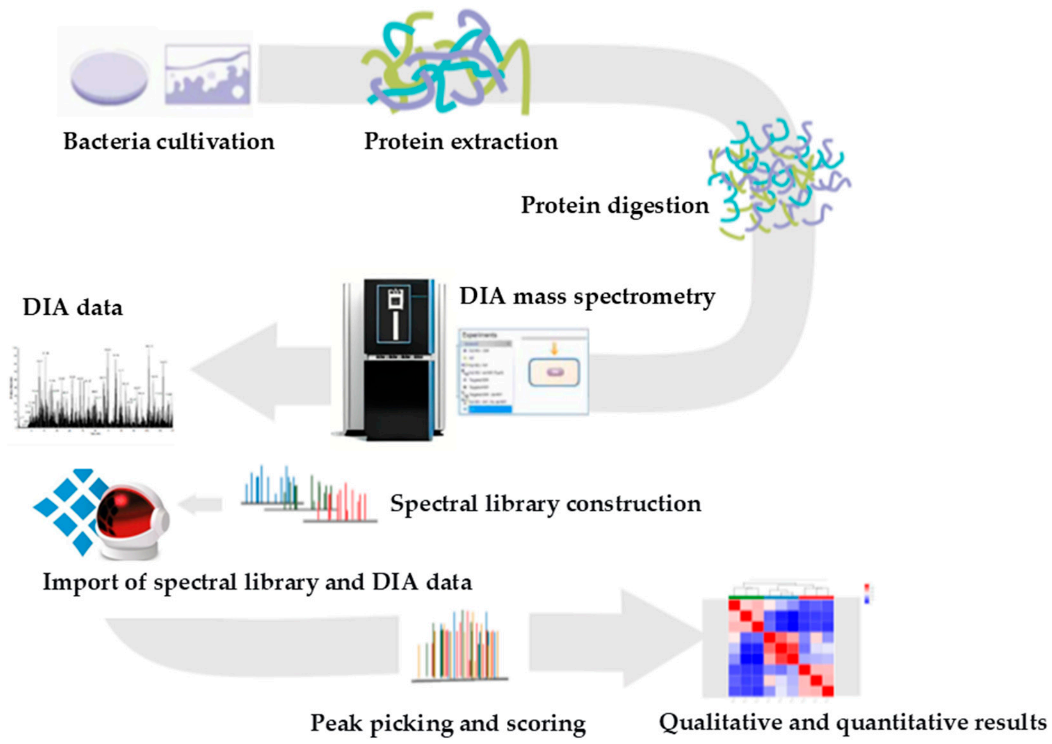

Figure S1. Schematic workflow of the Data-Independent Acquisition (DIA) project.

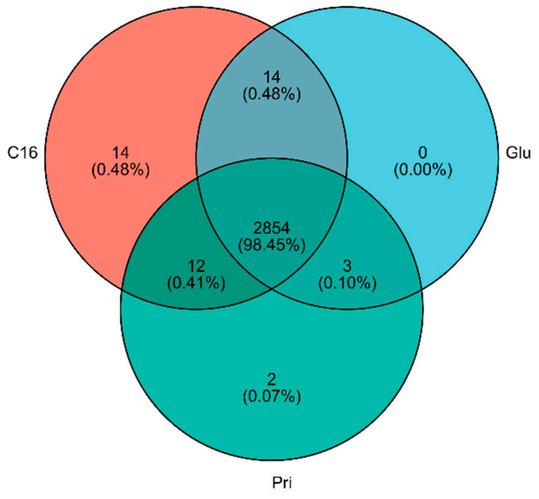

Figure S2. Venn diagram showing the overlap of identified proteins among the C_{16}, pristane (Pri) and glucose (Glu) treatments.

https://doi.org/10.3390/microorganisms14092100

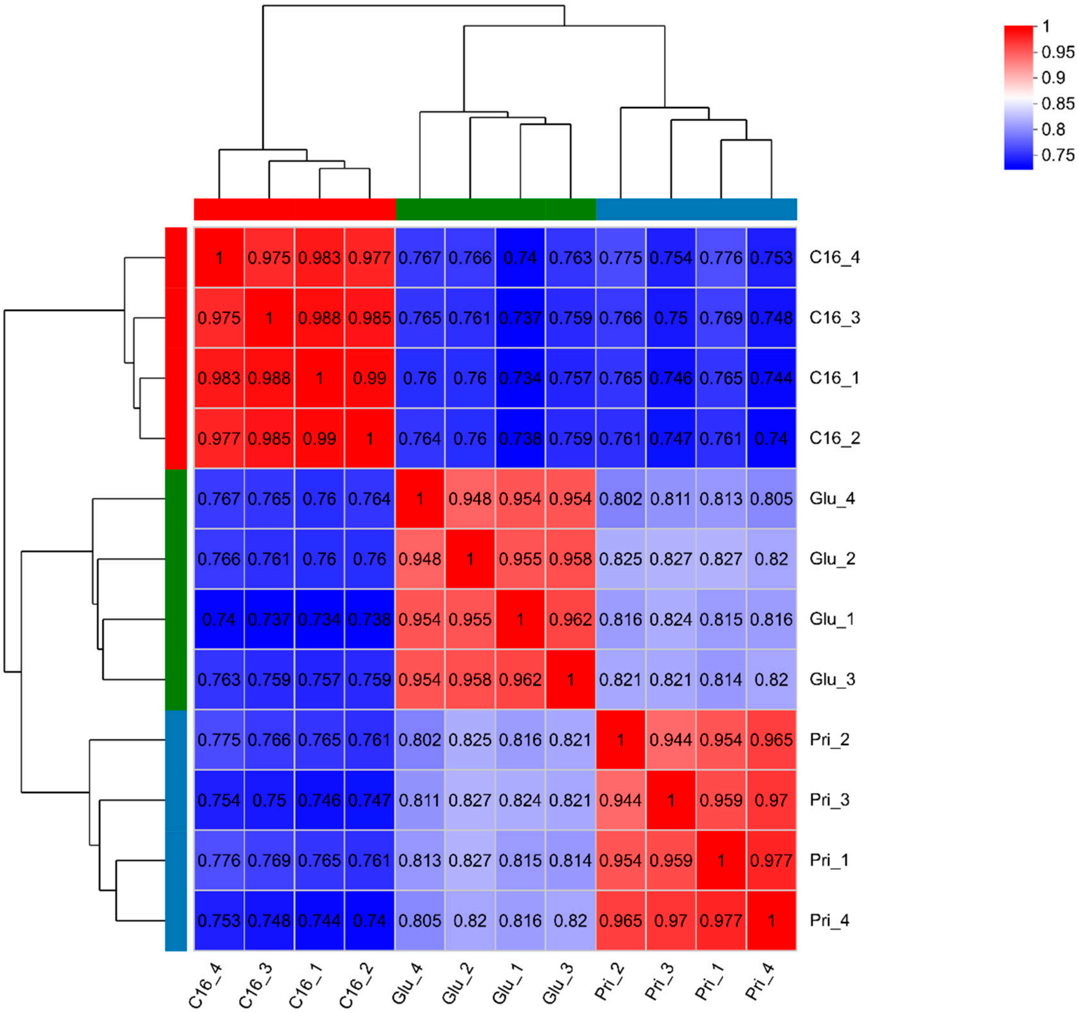

Figure S3. Correlation analysis of samples from the strain CN-3 under pristane (Pri), glucose (Glu), and C_{16} treatments. Biological replicates (1–4) are indicated for each group.

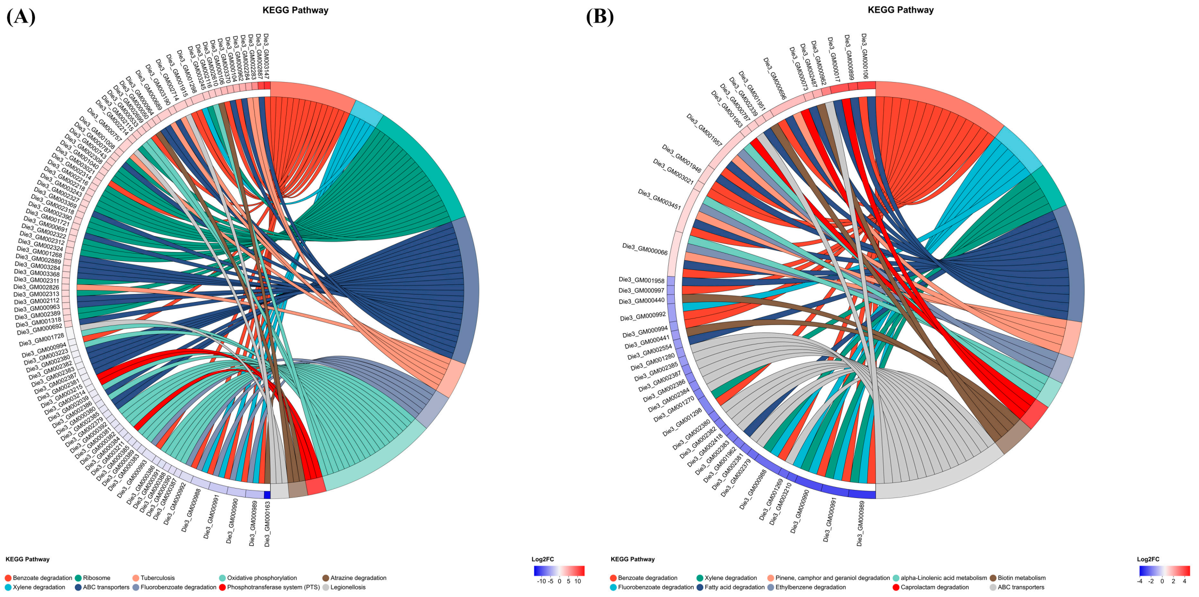

Figure S4. KEGG enrichment chord diagrams of DEPs in strain CN-3 for the treatment groups C_{16} vs Glu (A) and Pri vs Glu (B). The left semicircle represents the proteins, which are arranged from top to bottom along with their log_2FC values. The right semicircle displays the names of the enriched KEGG pathways and their corresponding z-scores. A positive z-score indicates that more upregulated than downregulated proteins are involved in the pathway, whereas a negative z-score indicates that more downregulated than upregulated proteins are involved.

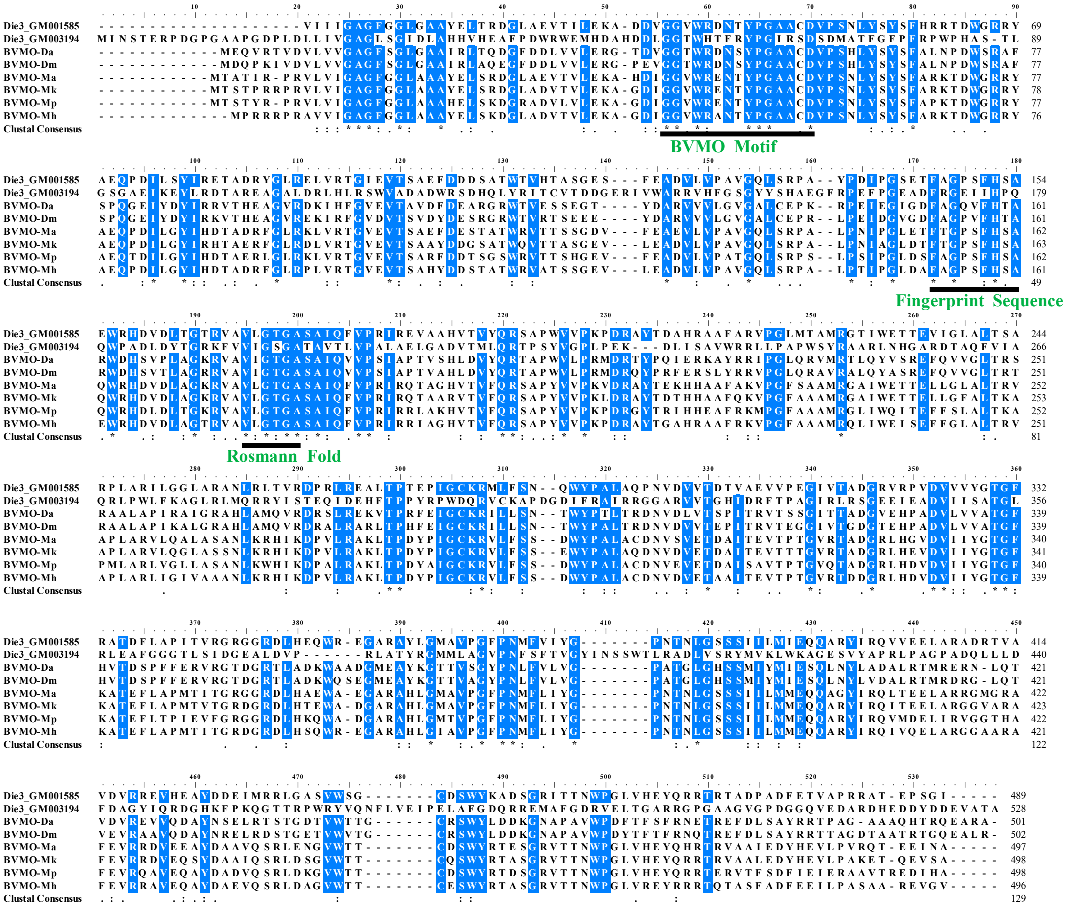

Figure S5. Multiple sequence alignment of different Baeyer-Villiger monoxygenases (BVMOs). Two BVMO homologous genes including Die3_GM001585 and Die3_GM003194 from CN-3, BVMO-Da: Baeyer-Villiger monoxygenases from *Dietzia aurantiaca*, BVMO-Dm: Baeyer-Villiger monoxygenases from *Dietzia maris*; BVMO-Ma: Baeyer-Villiger monoxygenases from *Mycobacterium asiaticum*, BVMO-Mk: Baeyer-Villiger monoxygenases from *Mycobacterium kubicae*, BVMO-Mp: Baeyer-Villiger monoxygenases from *Mycobacterium paraterrae*; BVMO-Mh: Baeyer-Villiger monoxygenases from *Mycobacterium helveticum*. Asterisks (*) and dots (.) represent identical and similar residues, respectively; double dots (:) indicate higher similarity.

Table S1. Differential expression proteins related to oxidation of C_{16} and pristane.

Pathways	Gene ID	Protein annotation	C_{16}/Glu	Pri/Glu	Pri/C_{16}
Alkane hydroxylation	Die3_GM000017	Alkane hydroxylase-rubredoxin fusion protein, AlkB	5.38	3.78	−1.60
	Die3_GM000104	Ferredoxin	3.40	1.65	−1.75
	Die3_GM000105	Cytochrome P450, CYP153	5.81	5.00	−0.81
	Die3_GM000106	Ferredoxin reductase	3.18	4.13	0.95
Subterminal oxidation	Die3_GM001585	Baeyer-Villiger monooxygenase	2.27	0.02	−2.25
	Die3_GM003194	Baeyer-Villiger monooxygenase	1.45	0.42	−1.03
	Die3_GM001424	Esterase	1.05	0.52	−0.53
Dehydrogenation of alcohols	Die3_GM001147	Alcohol dehydrogenase	1.56	1.23	−0.33
	Die3_GM001258	Alcohol dehydrogenase	−1.32	−0.10	1.22
	Die3_GM001489	Alcohol dehydrogenase	1.17	0.14	−1.03
	Die3_GM002334	Alcohol dehydrogenase	−1.13	−0.53	0.60
	Die3_GM002478	Alcohol dehydrogenase	−1.76	0.46	2.23
	Die3_GM002554	Alcohol dehydrogenase	−2.76	−1.20	1.56
	Die3_GM003062	Alcohol dehydrogenase	1.71	1.05	−0.66
Dehydrogenation of aldehydes	Die3_GM000241	Aldehyde dehydrogenase	−1.86	−0.65	1.21
	Die3_GM001027	Aldehyde dehydrogenase	1.43	0.21	−1.21
	Die3_GM002352	Aldehyde dehydrogenase	−0.12	1.52	1.64
	Die3_GM002477	Aldehyde dehydrogenase	−0.78	1.01	1.80

The numbers in "C_{16}/Glu", "Pri/Glu" and "Pri/C_{16}" represent $\log_2$FC of "C_{16} versus glucose", "pristane versus glucose" and "pristane versus C_{16}". Downregulated genes are marked with "−" values. All proteins listed in this table met the statistical significance criteria (adjusted $p < 0.05$).

Disclaimer/Publisher's Note: The statements, opinions and data contained in all publications are solely those of the individual author(s) and contributor(s) and not of MDPI and/or the editor(s). MDPI and/or the editor(s) disclaim responsibility for any injury to people or property resulting from any ideas, methods, instructions or products referred to in the content.

https://doi.org/10.3390/microorganisms14092100
